# The changing epidemiology of human leishmaniasis in the non-endemic country of Austria between 2000 to 2021, including a congenital case

**DOI:** 10.1371/journal.pntd.0011875

**Published:** 2024-01-10

**Authors:** Katharina Riebenbauer, Stefan Czerny, Maximilian Egg, Nikolaus Urban, Tamar Kinaciyan, Amélie Hampel, Luise Fidelsberger, Franz Karlhofer, Stefanie Porkert, Julia Walochnik, Alessandra Handisurya

**Affiliations:** 1 Department of Dermatology, Medical University of Vienna, Vienna, Austria; 2 Institute of Specific Prophylaxis and Tropical Medicine, Center for Pathophysiology, Infectiology and Immunology, Medical University of Vienna, Vienna, Austria; University of Washington, UNITED STATES

## Abstract

**Background:**

Leishmaniasis is caused by infection with intracellular protozoans of the genus *Leishmania*. Transmission occurs predominantly by the bite of phlebotomine sandflies, other routes, including congenital transmission, are rare. The disease manifests as either cutaneous, visceral or mucosal/mucocutaneous leishmaniasis. In recent years, changes in the epidemiological pattern have been reported from Europe.

**Principal findings:**

A total of 311 new and 29 published leishmaniasis cases occurring between 01/01/2000 and 12/31/2021 in Austria were collected and analyzed. These encompassed 146 cutaneous (CL), 14 visceral (VL), 4 mucosal, and 3 cases with concurrent VL and CL. In addition, asymptomatic infections, comprising 11 unspecified cases with *Leishmania* DNA detectable only in the blood and 162 cases with anti-*Leishmania* antibodies were reported. Particularly since 2016, the incidence of leishmaniasis has steadily risen, mainly attributable to increasing numbers of CL and cases with positive serology against *Leishmania* species, whereas the incidence of VL has slowly decreased. Analysis revealed that a shift in the causative species spectrum had occurred and that a substantial number of CL cases were caused by members of the *Leishmania donovani/infantum* complex. Simultaneous occurrence of VL and CL was identified in immunocompromised individuals, but also in a not yet reported case of an immunocompetent child after vertical transmission.

**Conclusions:**

The incidence of leishmaniasis has risen in the recent years. The numbers are anticipated to keep rising due to increasing human mobility, including travel and forced migration, growing reservoir host populations as well as expansion and dispersal of vector species caused by climate and habitat changes, urbanization and globalization. Hence, elevated awareness for the disease, including possible transmission in previously non-endemic regions and non-vector transmission modes, support of sandfly surveillance efforts and implementation and establishment of public health interventions in a One Health approach are pivotal in the global efforts to control and reduce leishmaniasis.

## Author summary

Leishmaniasis is a vector-borne infectious disease caused by intracellular parasites and affects 12–15 million people worldwide. The clinical presentation is variable, and the disease mainly manifests as cutaneous, visceral or mucosal forms. We collected 340 cases of leishmaniasis occurring in Austria over a period of 21 years and performed an analysis of trends to understand the epidemiology of the disease in a non-endemic Central European country. In addition, we report a rare case of concurrent visceral and cutaneous leishmaniasis which was vertically transmitted to an immunocompetent child. Our analyses revealed that the incidences of cutaneous leishmaniasis are rising in Austria and that a shift in the causative species spectrum has occurred. Due to increased population migration, travel activities of humans and animal hosts, growth and spread of the vector populations facilitated primarily by climate changes, a rising number of leishmaniasis cases can be anticipated in many endemic, but intriguingly also in non-endemic regions. The potential expansion of endemicity necessitates raising awareness towards the burden of this disease and provides an incentive to the global health community to continue their efforts in disease control.

## Introduction

Leishmaniasis, a multifaceted disease caused by intracellular protozoan parasites of the genus *Leishmania*, belongs to the group of “Neglected Tropical Diseases”, as classified by the World Health Organization (WHO). An estimated 12–15 million people worldwide are currently infected, with 0.7–1 million new cases occurring per year and up to one billion individuals at risk of infection [1].

Leishmaniasis presents a variety of symptoms, which are grouped into three main phenotypic disease forms, cutaneous (CL), visceral (VL) and mucosal (ML)/mucocutaneous (MCL) leishmaniasis [1–3]. The course and severity of infection depend on the virulence of the causative parasite strain and the immune status of the human host. At least 20 *Leishmania* species have been identified as pathogens of humans and are categorized into Old World or New World species according to their respective inhabited geographical regions. The parasites are transmitted as anthroponosis or zoonosis between human and animal hosts, such as dogs and rodents, through the bite of phlebotomine sandflies, *Phlebotomus* species in the Old World and *Lutzomyia* species in the New World [3]. Non-vector-based modes of transmission are less frequent and include blood transfusions, organ transplantations and intravenous drug use [2]. While vertical transmission is assumed to occur commonly in animals [4], in humans this mode is exceedingly rare, albeit sporadic cases of congenital VL have been reported [5,6].

The most important causative agents of the most common form, CL, are *Leishmania* (*L*.) *tropica*, *L*. *major*, *L*. *aethiopica*, and, less frequently, *L*. *infantum* and *L*. *donovani* in the Old World and *L*. *mexicana*, *L*. *braziliensis*, *L*. *guyanensis*, *L*. *panamensis* and *L*. *amazonensis* in the New World [3]. The most severe form, VL, is caused by *L*. *donovani* and *L*. *infantum* and usually fatal without adequate treatment due to severe anemia and immunosuppression, the latter facilitating secondary bacterial infections [7]. The third form, ML/MCL, is the most disfiguring form and predominantly occurs after infection with *L*. *braziliensis*. Other species that can cause ML/MCL comprise *L*. *mexicana*. *L*. *panamensis*, *L*. *guyanensis*, *L*. *amazonensis*, *L*. *major*, *L*. *tropica*, and *L*. *infantum*.

To date, about 100 countries throughout Asia, Africa, the Middle East, Central and South America, and the Mediterranean region are considered endemic for leishmaniasis [8,9]. In recent years, the global incidence of CL has steadily increased, whereas since 2012, the number of VL cases has decreased worldwide as a result of efforts in vector control and improvement of health care [8,10]. However, outbreaks of leishmaniasis still affect thousands of people inside and outside endemic countries, particularly in regions with armed conflicts [11–15]. The recrudescence of leishmaniasis in these regions as a consequence of human displacement, migration and urbanization impelled by armed conflicts, environmental disasters, economic circumstances or social pressure further bear the potential of introducing leishmaniasis to new geographical areas. In addition, the expansion of sandfly populations, supported by climate changes and evolving resistance to insecticides, and the revival of travel activities of humans and animal hosts after the COVID-19 pandemic may contribute to the dissemination of leishmaniasis.

In Austria, which is considered non-endemic for leishmaniasis [9], this disease is not notifiable, thus, the availability of data is limited. Two assumedly autochthonous VL cases with non-identified origin of infection were published in 1965 and 1989 [16,17], and since then a number of imported cases of CL and VL have been reported [18–29]. Moreover, a relatively high seroprevalence of anti-*Leishmania* antibodies was shown in several serological surveillance studies conducted in Austrian military personnel [30–33].

Herein, we report and summarize new and published cases of leishmaniasis occurring in Austria between 2000 and 2021, including a new case of rare congenital concurrent VL and CL in a child, in order to provide an overview of the epidemiology of this disease in a Central European country.

## Methods

### Ethics statement

This study was approved by the Ethics Committee of the Medical University of Vienna, Austria (EK2169/2021). Formal consent was not required from of the participants due to the retrospective nature of the study. Written informed consent was obtained from the parents of the child participant to be included in this study and for the images to be published.

### Study design and data sources

A systematic collection and analysis of leishmaniasis cases occurring in Austria during the period from January 1, 2000, to December 31, 2021, was retrospectively performed. Published data reporting cases of all forms of leishmaniasis occurring in Austria between 2000 and 2021 was obtained from publicly available online databases, such as PubMed, Google Scholar, Scopus and Semantic Scholar. However, reports of serological surveillance studies conducted in healthy, asymptomatic Austrian military personnel [30–33] were excluded, as these individuals were healthy and asymptomatic and could not be conflated with the study cohort, which consists of patients who had presented at their respective physicians with symptoms, pathologies or, at least, because of their fear to suffer from a disease, but not for research-based screening purposes. New, not yet reported data of leishmaniasis cases occurring in Austria between 2000 and 2021 was retrieved from the internal databases of the Institute of Specific Prophylaxis and Tropical Medicine of the Medical University of Vienna, which represents the national reference laboratory for parasitic infections in Austria, and from the electronic medical record system “Allgemeines Krankenhaus Informations Management (AKIM)” of the University Hospital of Vienna. The University Hospital Vienna is Europe’s fifth largest hospital by bed capacity and provides patient care not only to the 1.9 million inhabitants of the city of Vienna. It also covers the need of the population of the eastern part of Austria and, to a lesser part, of other national and international patients, but does not cover the entire country of Austria. Information on the disease form, the year of diagnosis, the sex, age, disease-related symptoms and afflicted body areas, co-morbidities and previous travel history of the patients as well as on the causative *Leishmania* species was obtained. Only cases, which had been confirmed by histological, molecular or serological diagnostic methods, were included.

Information regarding number and profile of the holiday and business trips in Austria and abroad made by the Austrian resident population, available only for the period 2003–2021, were obtained from the public database of the Statistik Austria [34].

The numbers of applications for international protection and refugee status according to the Geneva Convention and of applications for subsidiary protection submitted in Austria during the time period 2000–2021 were extracted from the statistical databases accessible on the official website of the Austrian Federal Ministry of the Interior [35].

Climate data on the annual average temperatures as well as the average temperatures in summer and winter in Austria for the period 1961–2021 were obtained from the public databases of the Zentralanstalt für Meteorologie und Geodynamik [36].

### Cohort and case description

A total of 340 published and new cases of leishmaniasis were collected. The individuals were subsequently categorized into three groups: (1) infections manifesting with disease, such as CL, VL and ML. (2) Asymptomatic infections with detectable *Leishmania* DNA in blood or positive serology. In addition, (3) borderline-positives, who were not considered as “cases” and, hence, were not subsumed into the study cohort, were compiled.

CL was defined by the presence of either *Leishmania* DNA or microscopically visible parasites in skin lesions. The cases classified as VL were either positive for *Leishmania* DNA in bone marrow and/or blood, or had previously been published as VL. In addition, two patients presenting with symptoms compatible of VL, consisting of sonographically confirmed spleno- and hepatomegaly, high fever, and pancytopenia, and specific *Leishmania* antibodies, as detected by ELISA and indirect immunofluorescence and verified by immunoblot, were considered a VL case. All ML cases had detectable *Leishmania* DNA in the mucosal tissues. Asymptomatically infected cases who had not been diagnosed with any above-mentioned forms of leishmaniasis, but had specific anti-*Leishmania* antibodies in the blood, detectable by ELISA or indirect immunofluorescence and confirmed by immunoblot, were considered serologically positive. Borderline-positives were defined as individuals, whose anti-*Leishmania* antibody results at screening could not be confirmed by a second serologic, immunoblot-based test and who had not developed any symptoms indicative of leishmaniasis over the available time period. If at a follow-up control visit the presence of specific anti-*Leishmania* antibodies could be verified by a confirmatory test, the case was regarded serologically positive. If serology remained without confirmation or if the patient was lost to follow-up, the individual was considered borderline-positive.

### Diagnostic approaches for parasite speciation

For molecular diagnostics of suspected leishmaniasis cases, the commercial *Leishmania* OligoC-Test (Coris BioConcept, Gembloux, Belgium), which detects the 18S ribosomal gene of 12 *Leishmania* species, was employed before 2017, whereas since 2017 all samples have been routinely screened using a multiplex real-time PCR [37]. This PCR allows the synchronous detection and identification of three *Leishmania* groups (the *L*. *donovani* complex, the *L*. *braziliensis* complex, and species other than these) in clinical samples by means of distinct melting temperatures, however, *L*. *tropica*, *L*. *major* and *L*. *mexicana* are not differentiated by this method. As in many cases the discrimination into these clinically relevant groups sufficed for medical treatment decisions, further speciation to the species level had not been performed. In the cases, where genotyping was applied, first a universal *Leishmania* PCR amplifying the internal transcribed spacer regions employing the LITSR/L5.8S primers [38] according to the protocol of Schönian et al. [39] was performed and the results confirmed by a second, independent PCR targeting the *Leishmania* heat-shock protein 70 gene [40]. The products of both PCRs were subsequently subjected to DNA sequencing and compared to the reference sequences from GenBank in order to fully resolve the causative agent down to the individual species level.

For serological analyses, two commercial tests were used: the Kalazar Detect Rapid Test for Visceral Leishmaniasis (InBios International, Inc., Seattle, USA) for qualitative detection of antibodies directed against the *L*. *donovani* rK39 antigen and the Leishmania Western Blot IgG (LDBio Diagnostics, Lyon, France), which is a qualitative test to detect IgG antibodies against the *L*. *infantum* antigen.

### Statistical analyses

An analysis of the data of all leishmaniasis cases as well as sub-analyses of the individual disease forms and of serologically positive cases were performed. Descriptive statistics were applied to the data. Continuous data are reported as mean ± standard deviation (SD) and categorical data as absolute and relative frequencies. The temporal trends of the absolute number of cases per year throughout the study period were assessed using a linear regression model employing the coefficient of determination (R-squared, R2) as the goodness-of-fit measure. The R2 values, used to express the proportion of the total variance of the response variable (absolute number of cases) with respect to the independent variables (year of diagnosis), were calculated by y = mx+b and obtained using Excel software. To investigate the association between the parameters “travel activities of the Austrian population” or “applications for asylum” with the variables “incidence trends of all leishmaniasis cases”, “incidence trends of each leishmaniasis form”, “seropositivity trends”, and each”*Leishmania* species causative of CL”, correlation analyses were performed employing the non-parametric Spearman’s rank correlation test. The correlation analyses were performed with GraphPad Prism, version 8.0, and the positive correlation given on a 0.1 to 1.0 scale. A correlation coefficient (r) in the range of 0.1–0.3 was considered weak, of 0.3–0.5 moderate, and of 0.5–1.0 strong. P-values (two-tailed) of < 0.05 were regarded statistically significant.

## Results

### Study cohort

**Fig 1:**
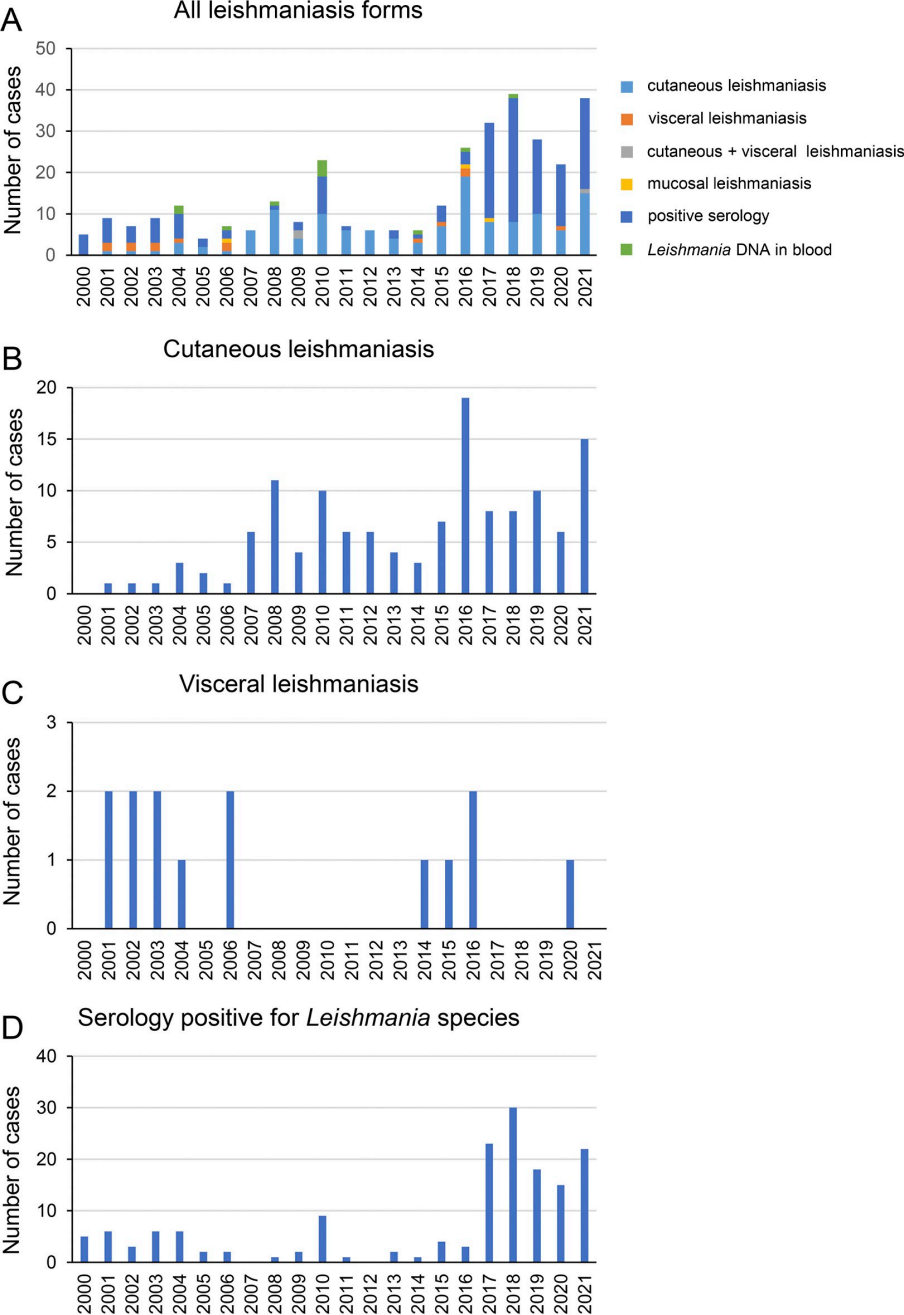
Incidences of (A) all leishmaniasis (B) cutaneous (C) visceral and (D) serologically positive cases diagnosed at the Medical University of Vienna, Austria, from 2000 to 2021.

A total of 340 published and new cases of leishmaniasis occurring in Austria between January 1, 2000, and December 31, 2021, were collected (Table 1). Since 2000 a steady increase in the incidences has been observed (Fig 1A). Compared to the decade 2000–2009, the numbers of leishmaniasis cases were 2.3-fold higher in the period 2010–2019. Particularly after 2016 the increase became markedly pronounced, reaching a peak in 2018 with 39 or 11.5% of all cases included in this study. While the COVID-19 pandemic suppressed the incidences in 2019 and 2020, the rising trend nevertheless continued, and the number of cases amounted to 60 already in the first two years of the current decade. The second highest peak in the entire observation period was noted in 2021 with 38 (11.2%) cases when the population resumed their pre-pandemic activities.

**Table 1:** Cases of leishmaniasis diagnosed at the Medical University of Vienna, Austria, 2000–2021.

	n (%)
infections manifesting with disease	167 (49.1)
cutaneous leishmaniasis	146 (42.9)
visceral leishmaniasis	14 (4.1)
visceral + cutaneous leishmaniasis	3 (0.9)
mucosal leishmaniasis	4 (1.2)
asymptomatic infections	173 (50.9)
*Leishmania* DNA in blood	11 (3.2)
positive serology	162 (47.7)
**Total**	**340 (100)**

### CL cases

During the 21-year period, a total of 146 CL cases were identified. 14 of these were reported in the period pertaining 2004–2010 [21] and, as the exact year of diagnosis had not been given in the publication, they were exempt from the incidence analysis, leaving a total of 132 CL cases (Fig 1B). The incidences showed a peak in 2008 followed by a decline until 2014. However, starting in 2015 an overall upward trend was noted, with the highest number of cases observed in 2016 and 2021. While 30 cases were recorded in 2000–2009, the numbers rose by 2.7-fold in 2010–2019 to 81 cases. In the following two years, the numbers continued to rise and 21 cases were already observed until the end of 2021.

The analysis of the 146 CL patients´ characteristics revealed a preponderance of the male sex at a ratio of 2.7 (Table 2). Primarily children, adolescents and adults up to the age of 30 were affected. Two patients had a concurrent, two an antecedent malignant disease, one patient was immunocompromised by human immunodeficiency virus (HIV). Of 89 patients, information regarding the localization of the skin lesions were available, revealing a predilection for the arms, followed by the legs and the head region.

**Table 2:** Characteristics of cutaneous leishmaniasis cases.

**Sex**	**n = 146 (100%)**
male	107 (73.3)
female	39 (26.7)
**Age** (years)	**n = 146 (100%)**
mean ± standard deviation	33.5 ± 22.7
range	1–96
**Age group** (years)	**n = 146 (100%)**
0–9	21 (14.4)
10–19	25 (17.1)
20–29	31 (21.2)
30–39	18 (12.3)
40–49	9 (6.2)
50–59	17 (11.6)
60–69	15 (10.3)
70–79	6 (4.1)
80–89	3 (2.1)
90–99	1 (0.7)
**Affected body areas**	**n = 89 (100%)**
head and neck	26 (29.2)
upper extremities	38 (42.7)
lower extremities	33 (37.1)
trunk, including axillae, buttocks	10 (11.2)

In the vast majority (93.8%) of the 146 CL cases, PCR was performed on material derived from the skin lesions. This method was highly sensitive with 131 (95.6%) of the 137 tested samples being positive, although for one sample the test result was not documented. In 19 cases, PCR was additionally performed from blood samples, which in two cases yielded a positive result for *Leishmania* DNA. One sample had derived from a 31-year-old female with multiple skin lesions located on both ankles and axilla, from which *L*. *braziliensis* was isolated from skin and blood after a travel to French Guiana. The second sample demonstrated the presence of *L*. *major* in skin and blood derived from a 54-year-old woman, of whom no further information was available. PCR was additionally performed on bone marrow samples of two CL patients, which were both negative. Histological evaluation of skin biopsies was performed in 85 (58.2%) of the 146 CL cases. Detection of *Leishmania* amastigotes in 40 of the specimens, however, revealed a lower sensitivity of 47.1% compared to PCR. Serological diagnostics were performed in 53 (36.3%) of the 146 CL cases and, interestingly, anti-*Leishmania* antibodies were detectable in 31 (58.5%) of these samples. Borderline-positive values by serology were obtained in 15.1%, negative results in 24.5% and in one (1.9%) case the result was unknown.

**Fig 2:**
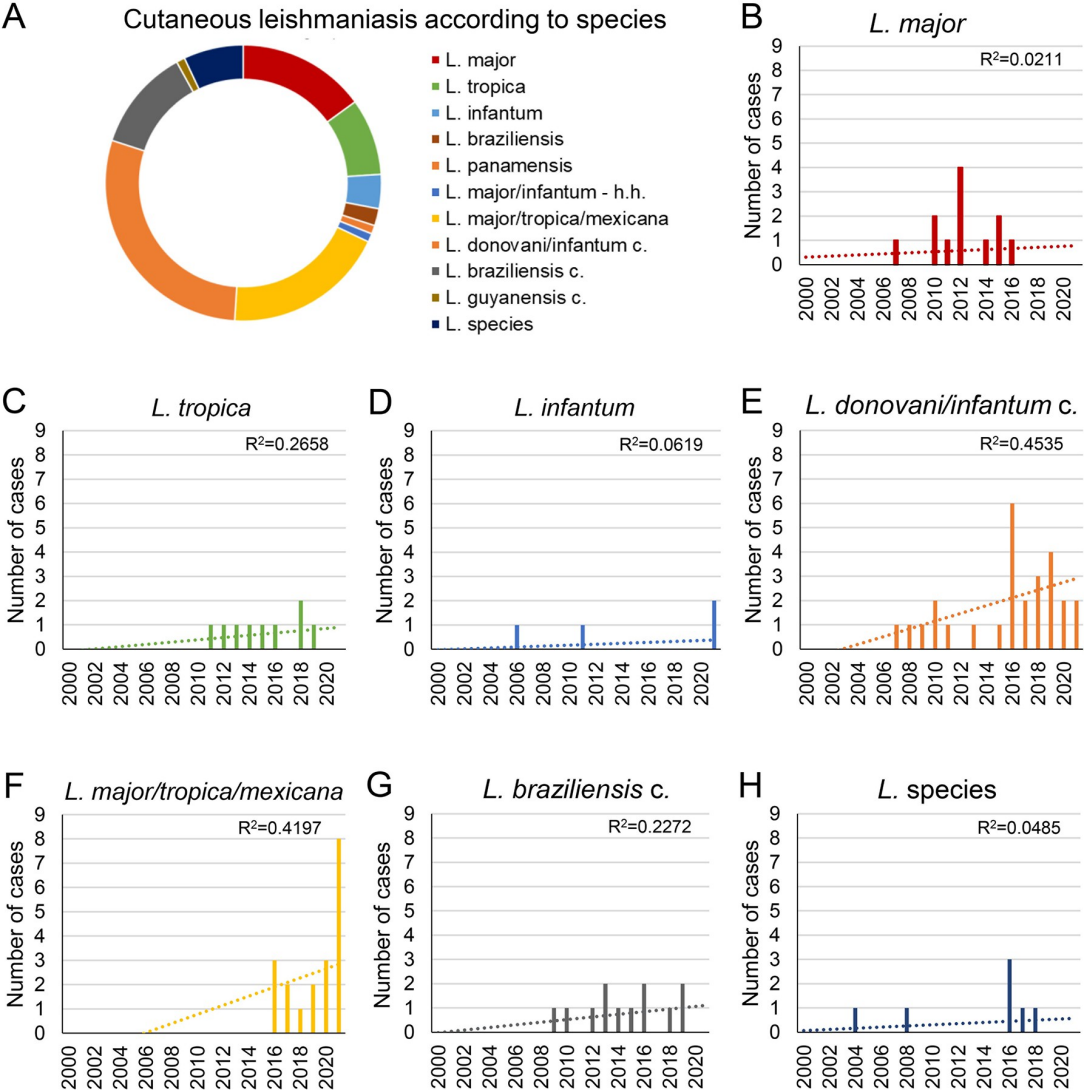
(A) Causative agents of cutaneous leishmaniasis cases according to species. Incidences of (B) Leishmania (L.) major, (C) L. tropica, (D) L. infantum, (E) members of the L. donovani/infantum complex (c.), (F) members belonging to the group L. major/tropica/mexicana, (G) members of the L. braziliensis c. and (H) unidentified L. species from 2000 to 2021. h.h., highly homologous; R2, coefficient of determination.

In 100 of the 146 CL cases information on the causative *Leishmania* species was available (Fig 2A and S1 Table). The most frequent species identified was *L*. *major* in 15 (15.0%) cases and its incidence has increased by 11-fold in 2010–2019 as opposed to 2000–2009 (Fig 2B). *L*. *tropica* was the second most common species, detected in 9 (9.0%) cases (Fig 2C). *L*. *infantum* was identified in 4 cases (4.0%) and two of these had occurred in 2021 (Fig 2D). *L braziliensis* and *L*. *panamensis* were rarely detected in two and in one case, respectively. A species with high sequence identities to *L*. *major* and *L*. *infantum* was isolated from a skin lesion on the knee of a 2-year-old Tunisian child in 2005. Particularly since 2016, increasing numbers of members of the *L*. *donovani*/*infantum* complex (Fig 2E) as well as of the group *L*. *tropica*/*major/mexicana* (Fig 2F) were identified as the causative agents. Members belonging to the *L*. *donovani*/*infantum* complex were the cause of 29 (29.0%), members of the group *L*. *tropica*/*major/mexicana* the cause of 19 (19.0%) cases. Members of the *L*. *braziliensis* complex were isolated in 12 (12.0%) cases (Fig 2G). One case was attributed to an unidentified member of the *L*. *guyanensis* complex [33], in 7 (7.0%) cases no further specification of the species was given (Fig 2H).

**Fig 3:**
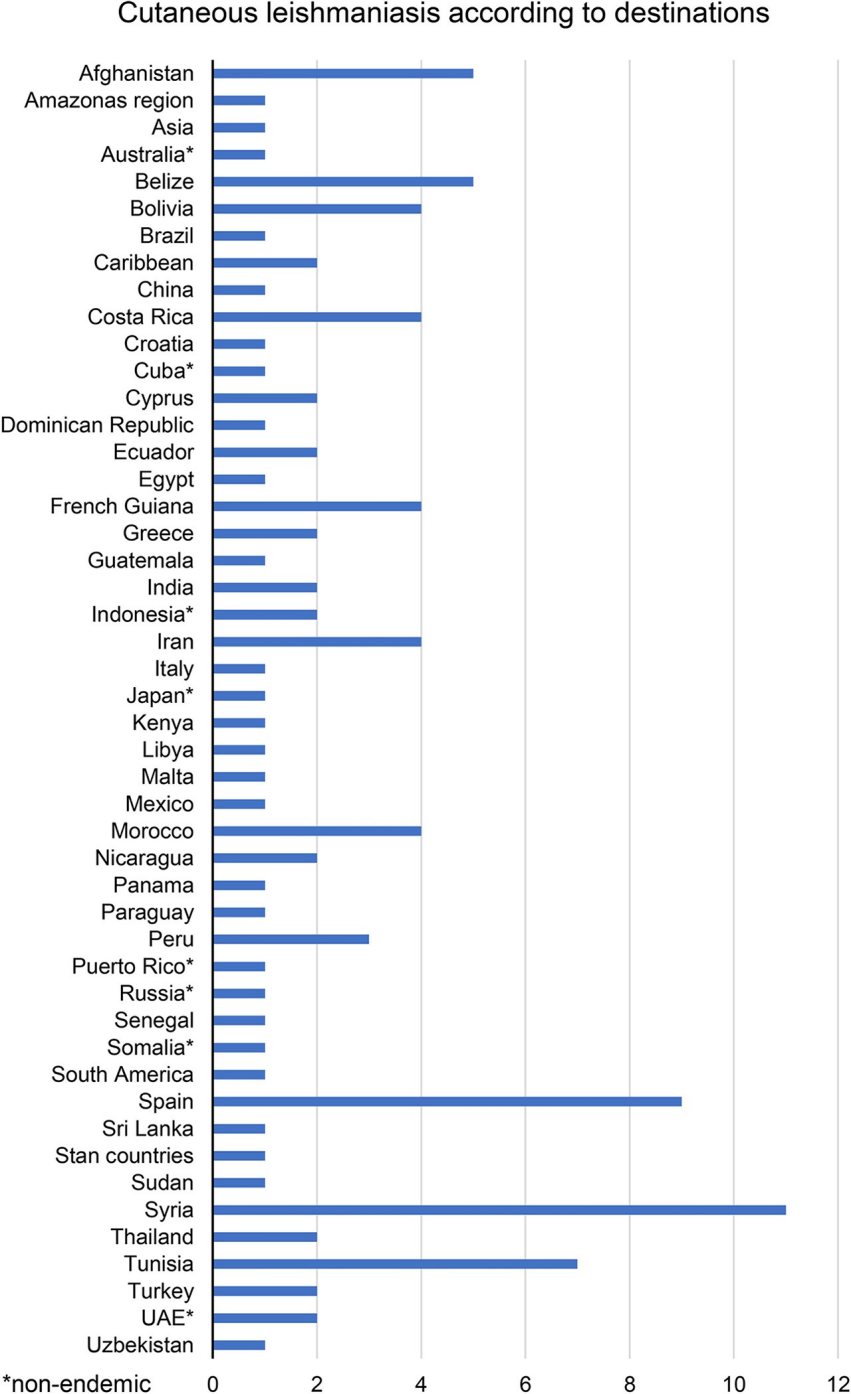
Countries and regions visited by patients with cutaneous leishmaniasis.

In 83 (56.8%) of the 146 CL cases, the travel history was available and 48 countries or regions were visited prior to development of disease (Fig 3). 39 (81.3%) of the destinations are considered endemic for CL according to the database of the WHO [9], the remaining non-endemic. The countries most frequently reported were Syria (13.3%), Spain (10.8%), Tunisia (8.4%), Afghanistan and Belize (each 6.0%).

### VL cases

In the study cohort, 14 cases of VL were identified. During the observation period the incidences slowly decreased, as demonstrated by 9 cases in 2000–2009 and 4 cases in 2010–2019 (Fig 1C). One case occurred in 2020 and from one case (excluded) the exact year of diagnosis could not be determined.

10 (71.4%) males and 4 (28.6%) females were afflicted, although the ratio of 2.5 was not as pronounced as compared to CL. The mean age of the afflicted individuals was 38.9±24.1 years, ranging from 0.1 to 73 years. One patient was immunosuppressed by HIV. Information on symptoms was not available from 4 patients. In the remaining 10 cases, pancytopenia was reported in all, splenomegaly in 8, fever in 5 and hepatomegaly in 4 of these patients. No cases of post-kala-azar dermal leishmaniasis were reported during the observation period.

For diagnostics PCR and/or histology was performed on bone marrow samples in 8 (57.1%) and 10 (71.4%) of the 14 VL cases, respectively. As expected, PCR showed a higher sensitivity (100%) compared to histology (50.0%). Serology was additionally performed in 12 (85.7%) VL cases, which yielded detectable anti-*Leishmania* antibodies in 10 (83.3%), borderline-positive results and negative results in one (8.3%) case each.

Information on the causative species was available in 7 VL cases of which 6 were caused by members of the *L*. *donovani/infantum* complex. Subsequent genotyping revealed *L*. *donovani* in 3 (42.9%) and *L*. *infantum* in one (14.3%) case. In one (14.3%) case the causative *Leishmania* species was not further specified.

Of 7 patients the travel history was reported and all had visited countries, such as France, Greece, Italy, Croatia, Spain, Kenya, and Thailand, which are listed as endemic for VL according to the WHO [9].

### Concurrent VL and CL, including a case of congenital leishmaniasis

In 3 cases, concurrent VL and CL were identified in the same patient. All cases were caused by members of the *L*. *donovani/infantum* complex.

In the first, new case, diagnosed in 2009, a 38-year-old man, immunocompromised due to HIV and renal transplantation, was affected. In this patient, *Leishmania* DNA was isolated from a skin lesion on the forehead as well as from bone marrow and blood, and parasites were histologically demonstrated in the bone marrow, but not in skin. In the second case, a 37-year-old, HIV-positive man, parasite DNA was detected in the generalized skin lesions, bone marrow, and blood. Furthermore, parasites were identified in skin, bone marrow, blood, gastric mucosa, and the biliary tract [22].

**Fig 4:**
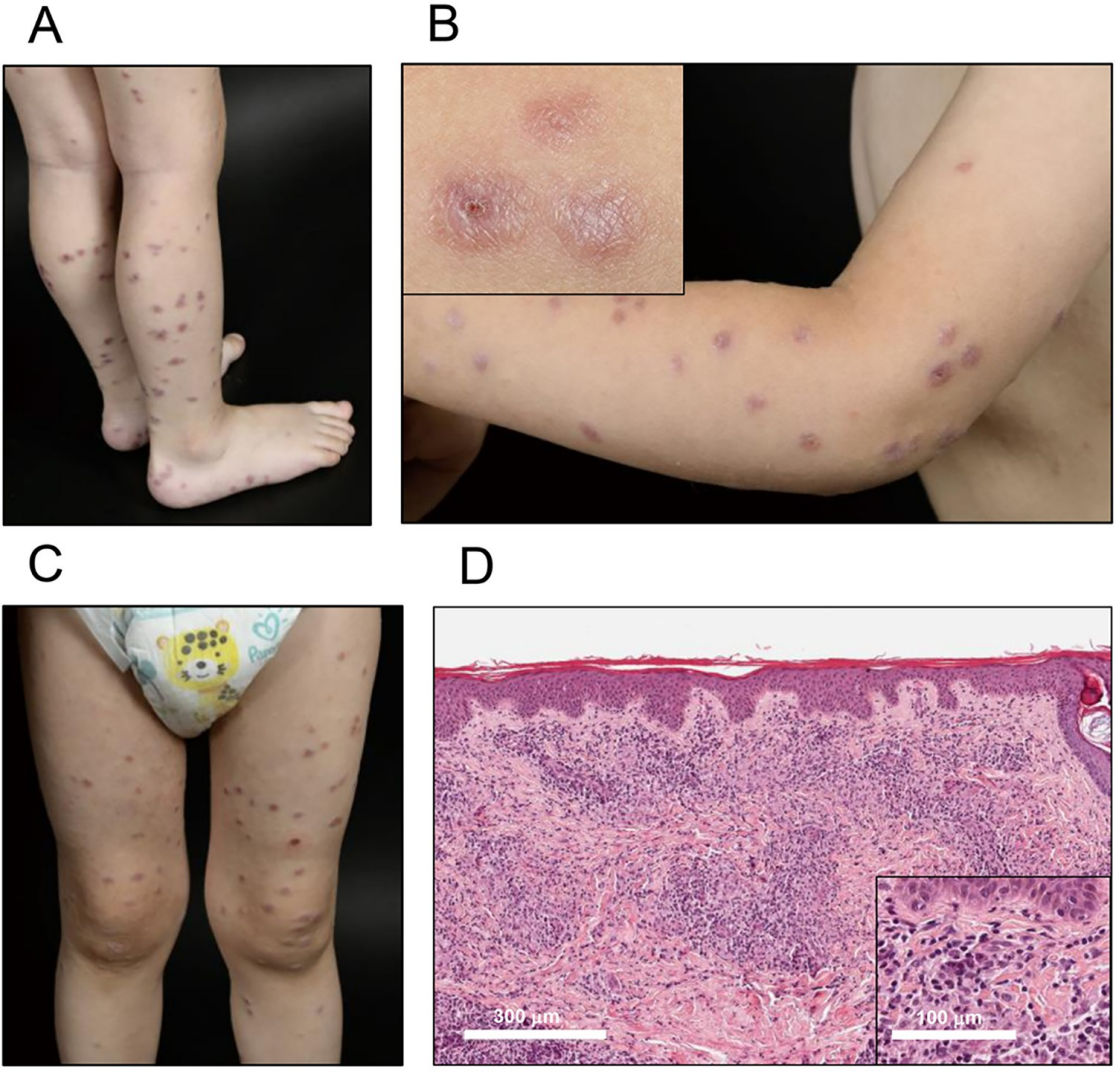
(A-C) Multiple erythematous, pruritic, painless plaques on the arms and legs of a two-year-old boy diagnosed with concurrent visceral and cutaneous leishmaniasis after congenital transmission of a member belonging to the *Leishmania donovani/infantum* complex. The insert shows the details of the skin lesions. (D). Hematoxylin-eosin stained tissue section of a skin lesion showing a lymphohistiocytic inflammatory infiltrate with interspersed plasma cells. The insert demonstrates the lack of morphologically visible pathogens.

Congenital leishmaniasis is rare, especially in European countries [5,41]. In 2021, a two-year-old boy presented to the dermatology outpatient clinic with pruritic skin lesions on the arms, legs, cheeks, and buttocks (Fig 4A–4C) that had been present for approximately six months. The skin lesions were accompanied by intermittent fever, weight loss, night sweats, and cervical lymphadenopathy. Routine blood examinations showed microcytic anemia, otherwise his blood counts, chemistry as well as immunological blood parameters were within normal reference ranges or without pathologies. Viral antibody testing performed in the child´s blood revealed no active viral infection, but IgG antibodies to Epstein Barr-, Varicella zoster-, Rubella-, Measles viruses and Parvovirus B19. Antibodies to HIV, Hepatitis B and Herpes simplex viruses were not detected. Abdominal ultrasound revealed splenomegaly. Histological evaluation of a biopsy taken from a skin lesion showed a lymphohistiocytic inflammatory infiltrate with interspersed plasma cells, but lack of morphologically visible pathogens (Fig 4D). The skin sections were additionally subjected to immunohistochemical staining for *Treponema pallidum*, Periodic Acid Schiff (PAS) staining for fungi and Ziehl-Neelsen staining for mycobacteria infection, which were all negative. Subsequent analysis of DNA, extracted from the paraffin-embedded skin tissue by PCR revealed the absence of bacterial and fungal infections, with the exception of *Malassezia restricta*. However, *Leishmania* DNA, more specifically DNA of a member of the *L*. *donovani/infantum* complex, was identified in the lesional skin tissues. There was no evidence of parasite DNA in the blood, but IgG against *Leishmania* species were detectable in the child´s serum. The boy was born in Austria and had never left the country, and abnormalities at birth or previous illnesses were denied by the parents. Screening of the patient’s asymptomatic family revealed anti-leishmanial IgG antibodies in the mother’s blood, whereas the remaining family members, including the older siblings, aged 6, 8 and 9 years were negative. Only the 5-year-old brother had a borderline-positive serology, but *Leishmania* DNA was not detectable in his blood. Upon questioning, the mother recalled traveling to the Romanian Black Sea coast, a region endemic for VL [9], in 2015 and 2016, before the birth of the patient and around the birth of his older brother, but denied having had any symptoms indicative of leishmaniasis. Based on the patient´s history and findings, a diagnosis of vertically transmitted concurrent VL and diffuse CL was made. The patient was treated with liposomal amphotericin B, which led to an improvement of the condition over the course of several months.

### ML/MCL cases

Mucosal involvement is rare in travel-related cases. Additional to two published cases [20,24], herein, we identified two new cases, one occurring in 2006 and one in 2016.

The ML/MCL cases were all males in their forties with a mean age of 45.3±4.3 years. Involvement of the nasal septum and the tongue were reported in two cases each. In all 4 cases diagnosis was made by PCR and histology, which yielded positive results in 100% and 75.0% of cases, respectively. Two cases were caused by *L*. *major* and two by members of the *L*. *donovani/infantum* complex. Both patients with *L*. *major* infections had extensive travel histories, including travels to Spain, Tunisia, Morocco, Egypt, India, Thailand, Indonesia, Argentina and Mexico. The two patients whose ML was caused by members of the *L*. *donovani*/*infantum* complex had been in either Italy or Croatia.

### Unspecified cases with *Leishmania* DNA in blood

In 11 cases, who were predominantly male (81.8%) and presented with a mean age of 33.2±18.9 years, range 3–60 years, *Leishmania* DNA was detected in the blood. In all cases skin lesions were not documented and PCR not performed on tissues derived from skin, bone marrow or other internal organs. Histological examination of bone marrow was done in a 7-year-old girl who had previously been in Romania, but yielded negative results. Genotyping of blood samples revealed a member of the *L*. *donovani/infantum* complex and a not further specified type in one case each, but additional information was lacking. Hence, the exact leishmaniasis form could not be identified in these patients.

### Cases with anti-*Leishmania* antibodies

Positive serological results were documented in 162 cases, but the appropriate leishmaniasis form could not be determined from the available patients´ records. Diagnostic PCR and histology from samples derived from skin, blood, bone marrow and other organs, performed in 64 (39.5%) of the cases, were all negative. During the observation period the incidences increased by 2.8-fold from 33 cases in 2000–2009 to 91 in 2010–2019 (Fig 1D), for one case the exact year of diagnosis could not be extracted. This trend presumably will rise further as 37 cases were already noted in the years 2020 to 2021. A marked increase was observed starting in 2017, which peaked in 2018 with 30 (18.5%) cases. The incidences slowly declined thereafter, but rose again in 2021, during which 22 cases (13.6%) were registered.

The male-to-female ratio was 1.3 and the mean age higher compared to CL and VL cases at 41.4±18.8 years with a range 1–94 years. The majority (79.6%) of cases were observed in the second to sixth decade, whereas children, adolescents and individuals aged above 70 years were less frequently affected. In 5 cases, a history of or concomitant malignancy and in 4 cases immunosuppression were recorded.

In 31 cases the travel history was given, and in total 37 countries and regions were visited, of which 27 were endemic [9] (S1 Fig). One case diagnosed in 2017, a 25-year-old woman who had never been abroad, but had reported a previous insect bite, was considered presumed autochthonous.

A total of 154 patients had a borderline-positive serological result, the value of which remains debatable.

## Discussion

Human leishmaniasis, particularly CL, appears to be on the rise in Europe and the Mediterranean region in recent years [10,42–52]. In Austria, an increasing number of cases have been observed in recent years, although exact data are not available due to the lack of a mandatory notification system or a central register. Aiming at providing an overview on the epidemiology of leishmaniasis in the non-endemic Central European country of Austria, we performed an analysis in an ample patient cohort, predominantly derived from the eastern part of Austria. However, as we could not capture all leishmaniasis cases occurring in the entire country, the prevalence of this disease may be severely underestimated. Our study revealed that the incidences, particularly of CL, are increasing. The underlying reasons for the rising incidences of leishmaniasis are presumably multifactorial and include human and animal movements and higher densities of vector and reservoir host species in various regions of the world.

**Fig 5:**
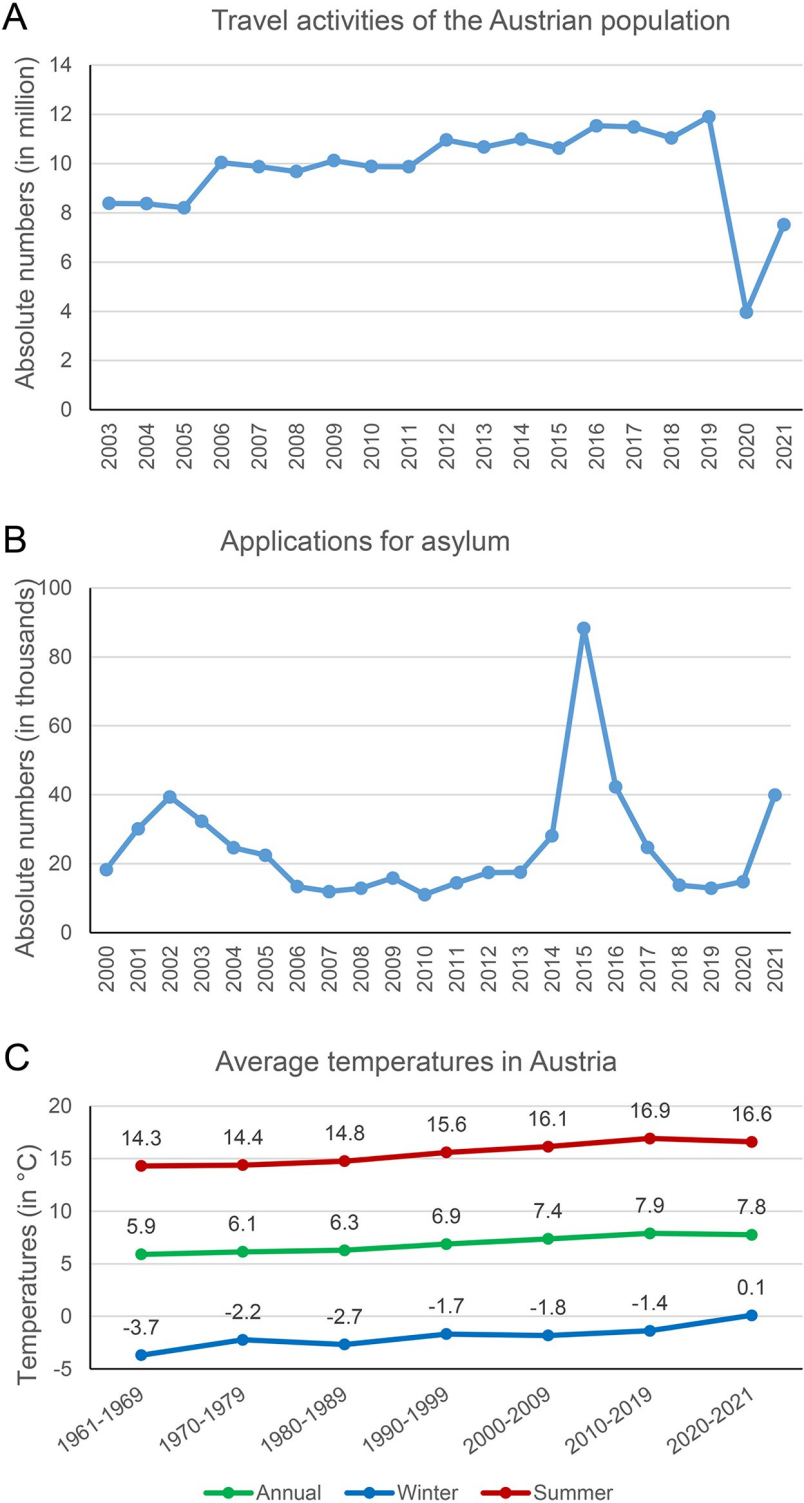
(A) Number of international holiday and business travel trips of the general Austrian population from 2003 to 2021. (B) Number of applications for international protection and refugee status and for subsidiary protection made in Austria from 2000–2021. (C) Annual average temperatures and average temperatures in summer and winter in Austria from 1961–2021.

Human movement is partly reflected by international travel activities. From 2003 to 2019, the number of holiday and business trips by the Austrian population (of roughly 9 million) has increased steadily (Fig 5A), with a registered average of 9.2 million travels per year in 2003–2009 to 10.2 million travels per year in 2010–2019 [34]. Among these, travel activities peaked at 11.5 and 11.9 million in 2016 and 2019, respectively. Mediterranean countries, such as Spain, Greece, Italy, and Croatia, are among the favorite travel destinations of Europeans, including the Austrian population, all of which are endemic for *Leishmania* species [9,53]. In addition, recent trends towards more experiential and adventurous travels drive travelers to more remote destinations and are often accompanied by higher infection risks due to more e.g. outdoor activities or sleeping in tents. Indeed, the travel activities of the Austrian general population correlated weakly with the herein reported incidence trends of all leishmaniasis cases (r = 0.1457) and of CL (r = 0.2665) and moderately with the trends observed for ML (r = 0.3970). However, the case numbers of ML in our cohort were low, and the associations statistically non-significant. The travel activities further showed a positive correlation with the incidence trends observed for certain species causative of CL. While strong correlations were observed for *L*. *tropica* (r = 0.6690; p = 0.002) and members of the *L*. *braziliensis* complex (r = 0.7229; p<0.001), the associations were moderate for members of the *L*. *donovani/infantum* complex (r = 0.3212) and weak for *L*. *major* (r = 0.2674), *L*. *tropica*/*major/mexicana* (r = 0.1358) and *L*. *infantum* (r = 0.2833). This indicates that intensive travel activities may partly account for the rising numbers of leishmaniasis in Austria.

However, human movement also encompasses forced migration and displacement. Displacement of people from leishmaniasis-endemic countries due to armed conflicts or natural disasters may represent a possible explanation for the introduction and dissemination of leishmaniasis, as observed for recently reported outbreaks [11–15]. In Austria, a marked increase in asylum applications was observed starting in 2014, with 28,064 applications, which rose to 88,340 applications in 2015 and 42,285 in 2016 (Fig 5B) [35]. More than half of these applications were submitted by individuals forced to leave their countries of origin, Afghanistan and Syria, both endemic to CL and VL [9]. While the numbers of asylum applications over the years did not correlate with the incidences of all leishmaniasis cases or of CL in particular, however, a strong association was found with the incidence trends of VL (r = 0.5270; p = 0.12). Weak correlations were observed with the incidences of ML (r = 0.1357) and seropositivity (r = 0.1740), as well as with *L*. *tropica*/*major/mexicana* (r = 0.1440), but not with other species causative for CL. Intriguingly, the forced human mobility seemed to have an impact on the pediatric population presenting with CL. A high proportion of the CL cases were between 1 and 19 years of age. In the 22 cases, where information on previously visited countries was available, the vast majority (81.8%) had originated from countries such as Afghanistan, Syria, Tunesia, Sudan and Somalia, all countries endemic for CL [9] and troubled by ongoing conflicts or economic crisis. This further unveils the problem that younger age groups are often immunologically not fully mature and more vulnerable to infections, including leishmaniasis. Early preventive measures, including increased screening and providing vaccination and adequate medical treatment to refugees, particularly when originating from endemic countries with less-developed health care, are urgently warranted to protect this vulnerable population and to decrease leishmaniasis in previously non-endemic areas.

Most *Leishmania* species are zoonotic. The zoonotic transmission to humans primarily from infected dogs, but also from rodents and other mammals play an important role in the occurrence and development of the disease. In this regard, canine leishmaniasis imported by travel and relocation has been documented in Austrian dogs [54]. While most infected dogs remain asymptomatic, they can still function as reservoirs for subsequent transmission to humans. Preventing infection in the reservoir host by prophylactic vaccination, use of repellents on dogs and their shelters, regular veterinary screening and supply of treatment to infected dogs may be difficult to achieve in resource-limited countries. However, these measures are worth establishing in global control plans to diminish leishmaniasis.

Furthermore, growing vector populations and dispersal of vectors to new habitats, facilitated by climate and habitat changes, flourishing international trade, urbanization and globalization may further contribute to high(er) incidences of leishmaniasis in many areas, including previously non-endemic regions. Sandfly populations are assumed to have been present in Central Europe since the end of the last glacial period, albeit in very small numbers [55]. In Austria, sandflies were first recorded in 2009 [56] and meanwhile, two species, *Phlebotomus mascittii* and *Phlebotomus simici* have been documented [57,58]. Vector competence for *Leishmania* species has been assumed, but still not proven for both species, thus it remains unclear, if these were involved in the transmission of the new presumably autochthonous case in 2017 and the published cases as of 1965 and 1989 [16,17]. It cannot be excluded that other, not yet detected sandfly species occur in Austria. Due to global warming, the conditions for sandfly development and reproduction have become more favorable, allowing for the vector populations to expand. Sandfly species have been detected at low densities also in more northern regions, up to Belgium and Germany [58,59]. According to simulations of global and regional climate models, temperatures across European land areas are predicted to rise this century by 1.2–8.5°C until 2071–2100, which is higher than the expected global average [60]. In Austria, the average annual and the average summer temperatures have increased from the 1960s to 2020s by 1.9°C and 2.3°C, respectively, and, notably, the average winter temperatures have surpassed the 0°C mark in 2020 (Fig 5C) [36]. Thus, without effective efforts and measures to halt global warming, this bears the potential of more European regions to become endemic for leishmaniasis vector species, and together with the existing and potentially growing reservoirs also endemic for the disease [59,61].

An important finding of our study was that in the recent years the *L*. *donovani/infantum* complex was increasingly detected as cause of CL in our patient population. Linear regression analyses of the trends over the past 21 years supported the positive relationship between this species complex and the observed increase. Formerly, the members of this complex have long been considered the causative agents of VL, and *L*. *infantum* specifically responsible for systemic and fatal VL in infants and immunocompromised individuals. However, *L*. *infantum* has only recently been recognized as cause for the development of CL [62]. Since then *L*. *infantum*-associated CL has been increasingly reported and in Europe, this entity is considered the only autochthonous cutaneous disease form [38]. Several *Phlebotomus* species have been incriminated as competent vectors of *L*. *infantum* and their geographical distribution is expected to extend north- and eastwards to Belgium, southern UK, Germany, Poland, Czechia, Slovakia, Romania, Moldova, Ukraine and also Austria throughout this century [59,61].

Numerous cases with anti-leishmanial antibodies or *Leishmania* DNA in the blood were detected in our study cohort and particularly since 2017 a sharp and sustained increase has been observed. A possible explanation for the higher frequency of serological diagnostics could be a heightened awareness or sensitivity among physicians regarding the disease. This could presumably have been elicited by the higher numbers of leishmaniasis patients physicians had encountered in the antecedent years, particularly since 2015, but could also be due to the increasing numbers of publications assessing leishmaniasis incidences in European countries [10,42–52]. In addition, serological surveillance studies conducted in asymptomatic Austrian military personnel, who had been deployed in the Kosovo, Syria, Lebanon or Bosnia and Herzegovina, revealed an unexpectedly high seroprevalence of antibodies against *Leishmania* [30–33]. Given the high rate of seropositivity in our cohort and in previous studies [32], the asymptomatic carriage of *Leishmania* species in the general population may be higher than assumed. This could have several important implications. First, although a large proportion of infected individuals may never demonstrate clinical manifestations, progression of disease still can develop later in life in the case of immunosuppression. Second, asymptomatic carriers of *Leishmania* species could serve as a reservoir of parasites. In a future scenario with expanding sandfly populations, these individuals may present a risk to the remaining population through vector-based transmission. Third, albeit human-to-human transmission is rare, non-vector-based transmission via blood products or donor organs remains possible, warranting the implementation of specific screening strategies for blood and organ banks. Fourth, congenitally transmitted leishmaniasis is rarely reported, as it is considered sporadic and implies an atypical mode of transmission. However, pregnant women may be more susceptible to recurrence or (re)activation of leishmaniasis due to altered immune responses. It is particularly important to note that the mother of the child described herein had been asymptomatic during her pregnancy and had never been diagnosed with leishmaniasis. Since congenital transmission in endemic countries is often indistinguishable from vector-borne transmission, reporting bias and underrepresentation can be assumed. In addition, the incubation period of vector-borne and congenital leishmaniasis can range from weeks to years [63], making it even more difficult to distinguish between these two forms.

## Conclusion

In summary, the pattern of leishmaniasis in Austria has changed over time. Therefore, physicians in endemic but also in non-endemic countries should be aware of the increasing burden of this disease and of the possible non-vector transmission modes to identify and timely treat the affected. Continuing the cooperation of human, animal, and environmental health partners to successfully implement and establish public health interventions in a One Health approach and the surveillance of sandfly distributions are pivotal in the global efforts to control and reduce leishmaniasis.

## Supporting information

S1 Table Causative species according to the year of diagnosis of patients with cutaneous leishmaniasis. (DOCX)

S1 Fig Destinations visited by Austrian patients with serologically confirmed antibodies to *Leishmania* species. (TIF)

S1 Data Supporting Dataset for Figures. (XLSX)
