# Supplementary material for: The changing epidemiology of human leishmaniasis in the non-endemic country of Austria between 2000 to 2021, including a congenital case
Source: PLoS Negl Trop Dis. 2024 Jan 10;18(1):e0011875. doi: 10.1371/journal.pntd.0011875 (PMC10805284; doi:10.1371/journal.pntd.0011875)
Supplement: S1 Table — (DOCX) [file pntd.0011875.s001.docx]

| **Species** | *L. major* | *L. tropica* | *L. infantum* | *L. braziliensis* | *L. panamensis* | *L. donovani/infantum* high homology | *L. donovani/infantum* complex | *L. major/tropica/mexicana* complex | *L. braziliensis* complex | *L. guyanensis* complex | *L.* species |
| --- | --- | --- | --- | --- | --- | --- | --- | --- | --- | --- | --- |
| **Year** |  |  |  |  |  |  |  |  |  |  |  |
| 2000 |  |  |  |  |  |  |  |  |  |  |  |
| 2001 |  |  |  |  |  |  |  |  |  |  |  |
| 2002 |  |  |  |  |  |  |  |  |  |  |  |
| 2003 |  |  |  |  |  |  |  |  |  |  |  |
| 2004 |  |  |  |  |  |  |  |  |  |  | 1 |
| 2005 |  |  |  |  |  | 1 |  |  |  |  |  |
| 2006 |  |  | 1 |  |  |  |  |  |  |  |  |
| 2007 | 1 |  |  |  |  |  | 1 |  |  |  |  |
| 2008 |  |  |  | 2 |  |  | 1 |  |  |  | 1 |
| 2009 |  |  |  |  |  |  | 1 |  | 1 |  |  |
| 2010 | 2 |  |  |  | 1 |  | 2 |  | 1 |  |  |
| 2011 | 1 | 1 | 1 |  |  |  | 1 |  |  |  |  |
| 2012 | 4 | 1 |  |  |  |  |  |  | 1 |  |  |
| 2013 |  | 1 |  |  |  |  | 1 |  | 2 |  |  |
| 2014 | 1 | 1 |  |  |  |  |  |  | 1 |  |  |
| 2015 | 2 | 1 |  |  |  |  | 1 |  | 1 |  |  |
| 2016 | 1 | 1 |  |  |  |  | 6 | 3 | 2 |  | 3 |
| 2017 |  |  |  |  |  |  | 2 | 2 |  |  | 1 |
| 2018 |  | 2 |  |  |  |  | 3 | 1 | 1 |  | 1 |
| 2019 |  | 1 |  |  |  |  | 4 | 2 | 2 |  |  |
| 2020 |  |  |  |  |  |  | 2 | 3 |  |  |  |
| 2021 |  |  | 2 |  |  |  | 2 | 8 |  |  |  |
| n.a.* | 3 |  |  |  |  |  | 2 |  |  | 1 |  |

*n.a; not available
