# Supplementary figures and images for: The changing epidemiology of human leishmaniasis in the non-endemic country of Austria between 2000 to 2021, including a congenital case
Source: PLoS Negl Trop Dis. 2024 Jan 10;18(1):e0011875. doi: 10.1371/journal.pntd.0011875 (PMC10805284; doi:10.1371/journal.pntd.0011875)

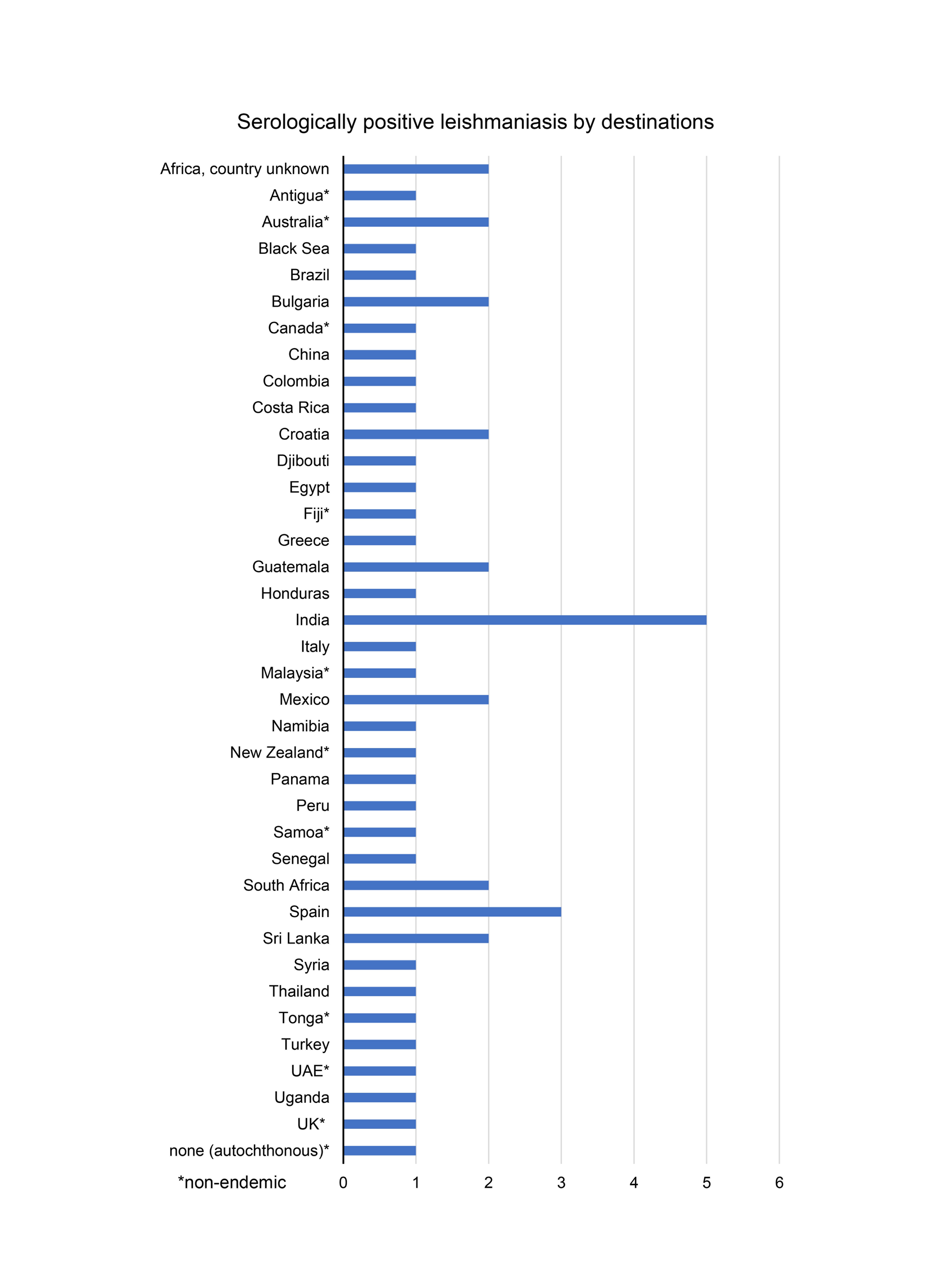

Supplement: S1 Fig — (TIF) [file pntd.0011875.s002.tif]
